# Supplementary material for: MetaRibo-Seq measures translation in microbiomes
Source: Nat Commun. 2020 Jun 29;11:3268. doi: 10.1038/s41467-020-17081-z (PMC7324362; doi:10.1038/s41467-020-17081-z)
Supplement: Supplementary file 10 — Supplementary Data 7 [file 41467_2020_17081_MOESM10_ESM.zip › File2/Confidence_High_Taxonomy/356757_out.krona.html]

Javascript must be enabled to view this page.

members
magnitude
magnitudeUnassigned
count
unassigned
taxon
rank

356757\_out

77

superkingdom
77
2

phylum
77
976

200643
class
77

171549
77
order

family
77
815

genus
77
816

1262739
species

SRS148159\_contig\_number\_49754
1

73

SRS011084\_contig\_number\_29120SRS012902\_contig\_number\_40SRS013098\_contig\_number\_28176SRS013158\_contig\_number\_1245SRS013215\_contig\_number\_128SRS014459\_contig\_number\_37238SRS014613\_contig\_number\_9597SRS014736\_contig\_number\_3818SRS015890\_contig\_number\_20075SRS015960\_contig\_number\_11717SRS016095\_contig\_number\_22668SRS016132\_contig\_number\_1612SRS017433\_contig\_number\_14568SRS017521\_contig\_number\_48908SRS017622\_contig\_number\_208SRS018656\_contig\_number\_11856SRS018817\_contig\_number\_contig-100\_455.131452SRS018888\_contig\_number\_2888SRS018984\_contig\_number\_27203SRS019397\_contig\_number\_12476SRS021484\_contig\_number\_26696SRS023346\_contig\_number\_2504SRS023829\_contig\_number\_589SRS023971\_contig\_number\_979SRS024075\_contig\_number\_9462SRS024132\_contig\_number\_45342SRS024388\_contig\_number\_14116SRS043411\_contig\_number\_1696SRS043701\_contig\_number\_2665SRS046712\_contig\_number\_2954SRS048981\_contig\_number\_6458SRS049402\_contig\_number\_8888SRS051610\_contig\_number\_9252SRS053214\_contig\_number\_20527SRS053573\_contig\_number\_2107SRS055966\_contig\_number\_924SRS057717\_contig\_number\_5763SRS063127\_contig\_number\_2553SRS063190\_contig\_number\_14324SRS063985\_contig\_number\_18319SRS064276\_contig\_number\_28513SRS074964\_contig\_number\_5285SRS075021\_contig\_number\_6188SRS075773\_contig\_number\_28474SRS075878\_contig\_number\_2165SRS077194\_contig\_number\_21736SRS077335\_contig\_number\_9056SRS077589\_contig\_number\_6706SRS077730\_contig\_number\_17295SRS098644\_contig\_number\_29140SRS098655\_contig\_number\_10269SRS098881\_contig\_number\_4868SRS104036\_contig\_number\_1763SRS1041039\_contig\_number\_7418SRS104311\_contig\_number\_15016SRS104485\_contig\_number\_1489SRS104636\_contig\_number\_contig-100\_1068.155036SRS1054928\_contig\_number\_6034SRS1055069\_contig\_number\_5472SRS140492\_contig\_number\_29656SRS140513\_contig\_number\_4997SRS142599\_contig\_number\_31061SRS143417\_contig\_number\_15846SRS143598\_contig\_number\_13651SRS144362\_contig\_number\_35305SRS144506\_contig\_number\_11778SRS146812\_contig\_number\_40052SRS147022\_contig\_number\_1968SRS147139\_contig\_number\_12475SRS147272\_contig\_number\_785SRS147653\_contig\_number\_2560SRS893279\_contig\_number\_2344SRS971275\_contig\_number\_42034
species
817

246787

SRS143342\_contig\_number\_3216
species
1


SRS1055043\_contig\_number\_10091
species
1
329854

1

SRS018541\_contig\_number\_1064
species
821
